# Supplementary material for: Adult fibroblasts use aggresomes only in distinct cell-states
Source: Sci Rep. 2022 Sep 2;12:15001. doi: 10.1038/s41598-022-19055-1 (PMC9440096; doi:10.1038/s41598-022-19055-1)
Supplement: Supplementary file 1 — Supplementary Information 1. [file 41598_2022_19055_MOESM1_ESM.pdf]

Supplementary Figures

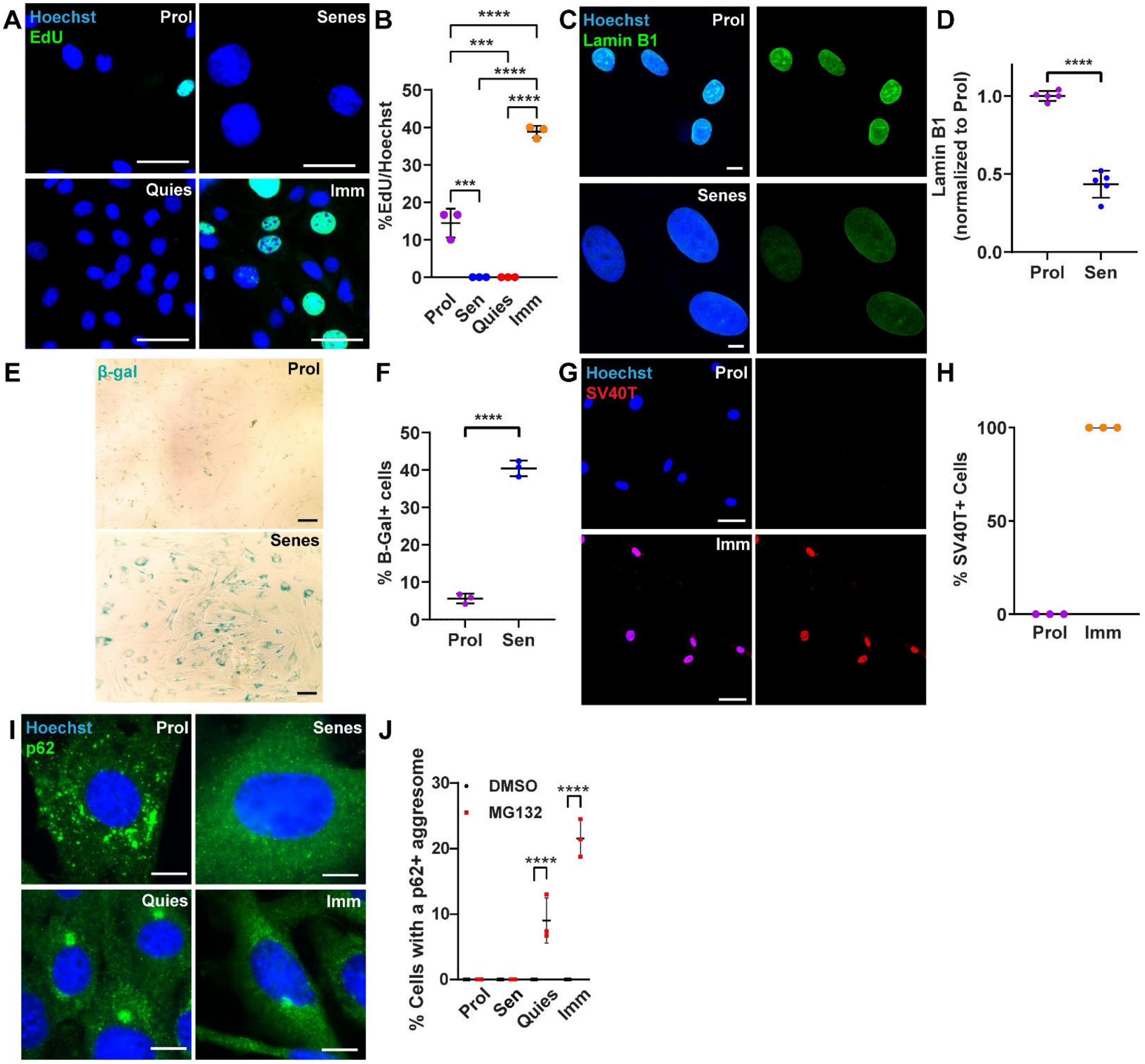

Figure S1 – A-B) Proliferating (purple), senescent (blue), quiescent (red), and immortalized (orange) fibroblasts were pulsed with EdU for 1 hour at 37°C prior to being fixed and stained to visualize nuclei (Hoechst; blue) and EdU (green). Cells were analyzed to identify the proportion of cells that were EdU+ (N=3; Two-way ANOVA with post-hoc Tukey's test; mean ± SD). C-D) Proliferating (purple) and senescent (blue) fibroblasts were fixed and immunostained for Lamin B1 (green) and stained for nuclei (Hoechst; blue). Samples were analyzed for relative intensity of Lamin B1 (N=3; Student's t-test; mean ± SD). E-F) Brightfield image of proliferating (purple) and senescent (blue) fibroblasts that were fixed and stained to visualize β-galactosidase activity (β-gal; green).

Samples were analyzed for the proportion of cells positive for β-galactosidase activity (N=3; Student's t-test; mean ± SD). G-H) Proliferating and immortalized fibroblasts were fixed and immunostained for SV40T (red) and stained for nuclei (Hoechst; blue). Samples were analyzed for the percentage of cells positive for SV40T (N=3; mean ± SD). I-J) Proliferating, senescent, quiescent, and immortalized fibroblasts were treated with 0.1% DMSO or 10 µM MG132 for 8 hours prior to fixation and immunostaining for p62 (green), and nuclei (Hoechst; blue). Samples were imaged, and subsequently analyzed for the proportion of cells forming aggresomes (N=3; Two-way ANOVA with post-hoc Tukey's test; mean ± SD). Scale bars, 50 µm (A, E, G), 10 µm (C, I). ***p<0.001, ****p<0.0001.

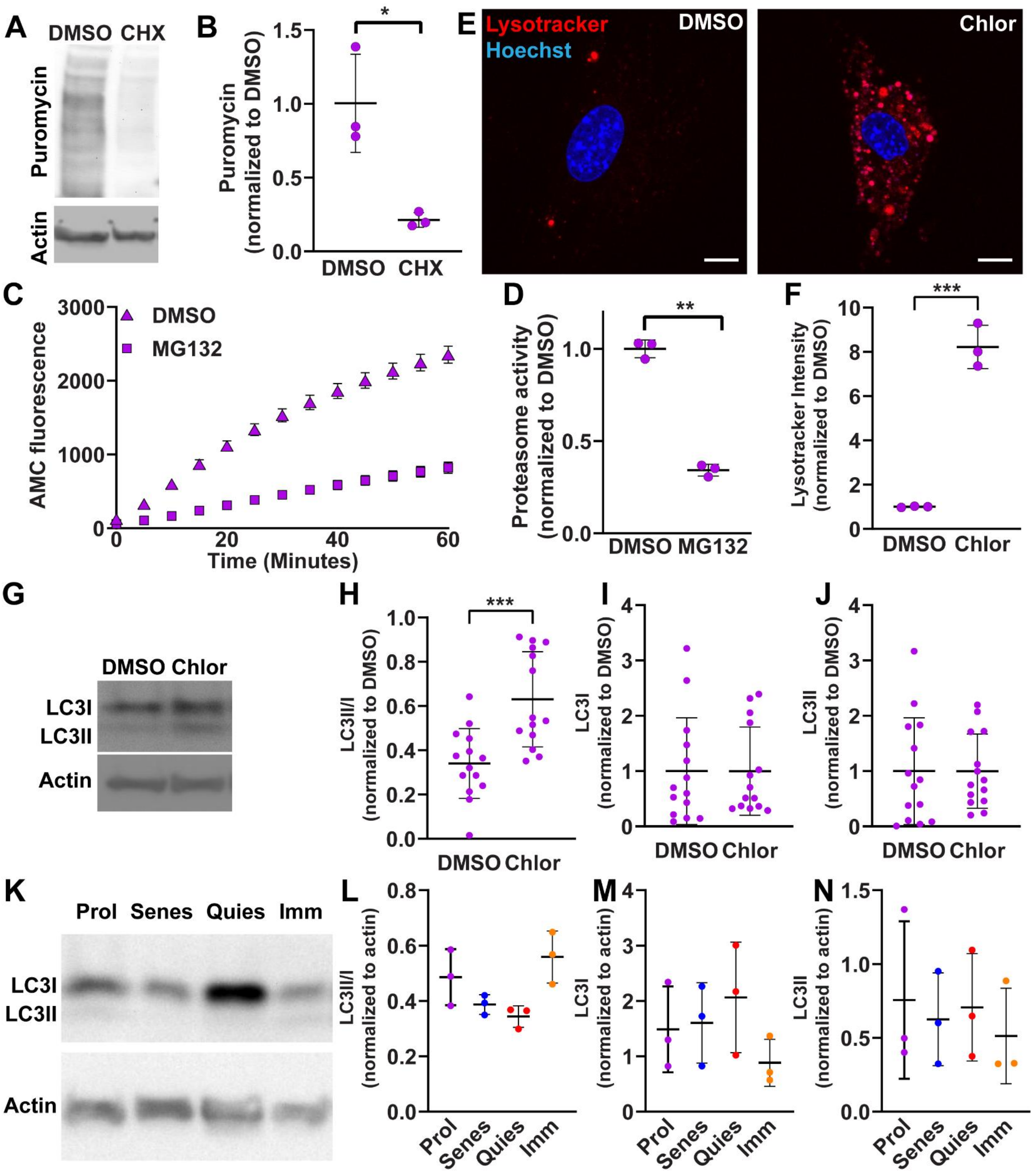

Figure S2 – A-B) Proliferating fibroblasts were treated with 10 µM cycloheximide or 0.1% DMSO for 10 minutes prior to addition of 10 µg/mL puromycin and then protein extraction and analysis of puromycin incorporation and actin expression by western blot. Samples were analyzed for relative puromycin incorporation relative to actin.

Uncropped blots are provided in Figure S4D-E (N=3; Student's t-test; mean ± SD). C-D) Proliferating fibroblast protein lysates were prepared and treated with either a control dose of 1% DMSO or 100 µM MG132 and then analyzed for relative levels of proteasome activity by measuring AMC fluorescence as a function of time. D displays normalized AMC accumulation at 60 minutes for each sample (N=3; Student's t-test; mean ± SD). E-F) Proliferating fibroblasts were treated with either 0.1% DMSO or 1 µM chloroquine for 24 hours and then stained and analyzed for lysosome content (Lysotracker; red) and nuclei (Hoechst; blue) (N=3; Student's t-test; mean ± SD). G-J) Proliferating fibroblasts were treated with either 0.1% DMSO or 1 µM chloroquine for 24 hours and analyzed by western blot for LC3 and actin levels (N=3; Two-way ANOVA with post-hoc Tukey's test; mean ± SD). K-N) Proliferating, senescent, quiescent, and immortalized fibroblasts were analyzed by western blot for LC3 and actin levels (N=3; Two-way ANOVA with post-hoc Tukey's test; mean ± SD). Scale bars, 10 µm. *p<0.05, **p<0.01, ***p<0.001.

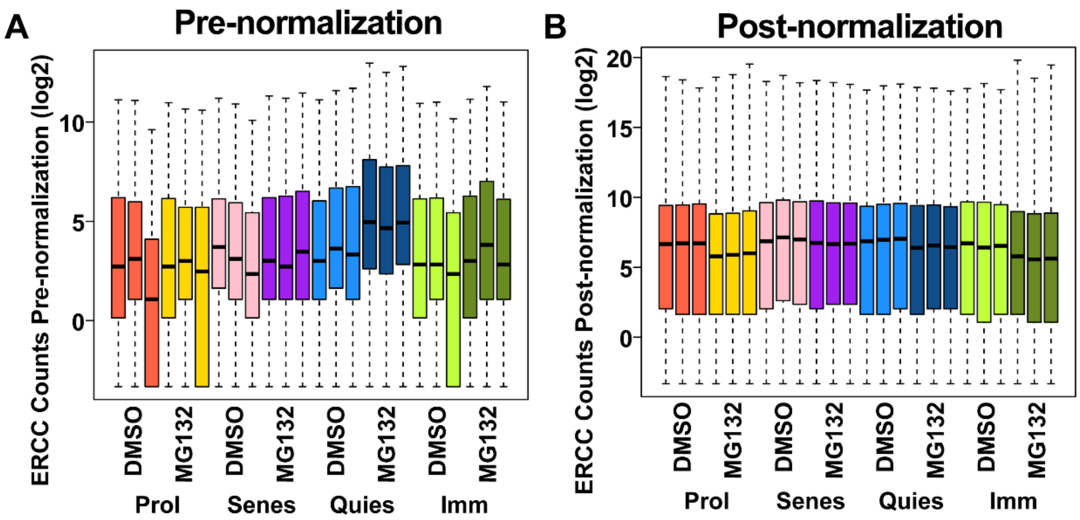

Figure S3 – A-B) ERCC spike-in counts relative to other transcripts in proliferating (prol), senescent (senes), quiescent (quies), and immortalized (imm) fibroblasts treated with DMSO or MG132 pre-ERCC normalization (A) or post-ERCC normalization (B). All RNA sequencing analyses shown are analyses performed using the post-normalized data.

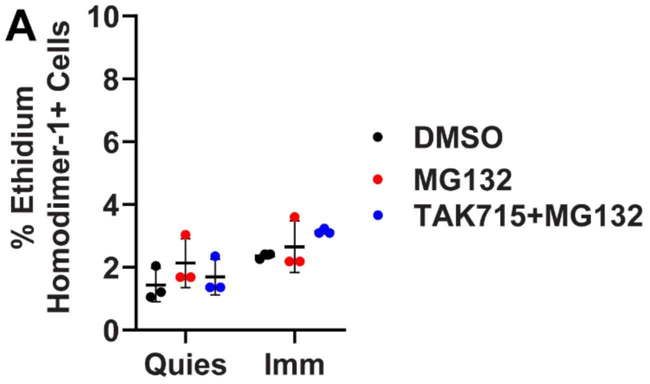

Figure S4 – A) Quiescent and immortalized fibroblasts were treated with 50 µM TAK-715 or 1% DMSO for 24 hours, then additionally treated with 10 µM MG132 for 7 hours still in the presence of TAK-715 or DMSO and then analyzed for the proportion of dead cells by labeling with Hoechst and ethidium homodimer-1.

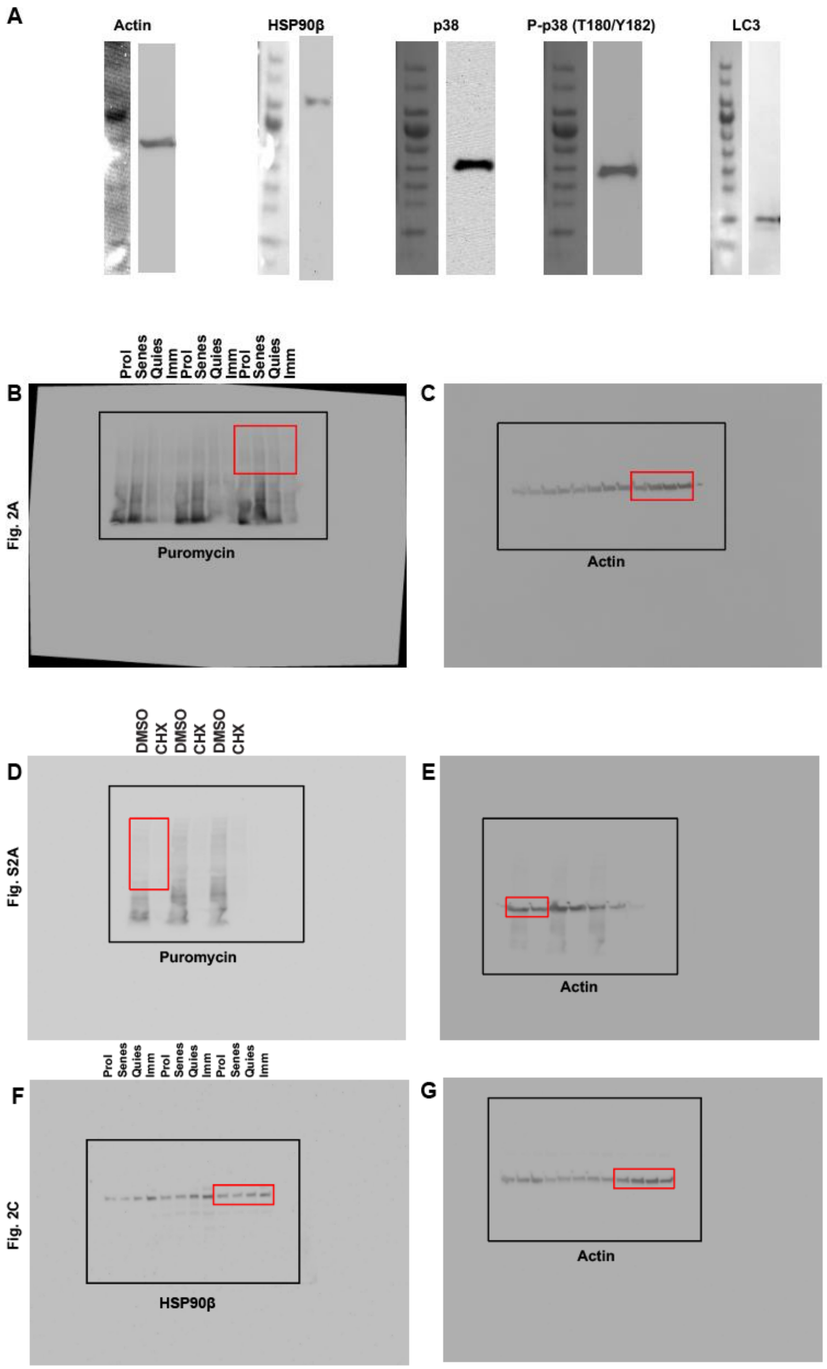
A
Actin
HSP90β
p38
P-p38 (T180/Y182)
LC3
Prol Senes Quies Imm Prol Senes Quies Imm Prol Senes Quies Imm
B
Fig. 2A
Puromycin
C
Actin
DMSO CHX DMSO CHX DMSO CHX
D
Fig. S2A
Puromycin
E
Actin
Prol Senes Quies Imm Prol Senes Quies Imm Prol Senes Quies Imm
F
Fig. 2C
HSP90β
G
Actin

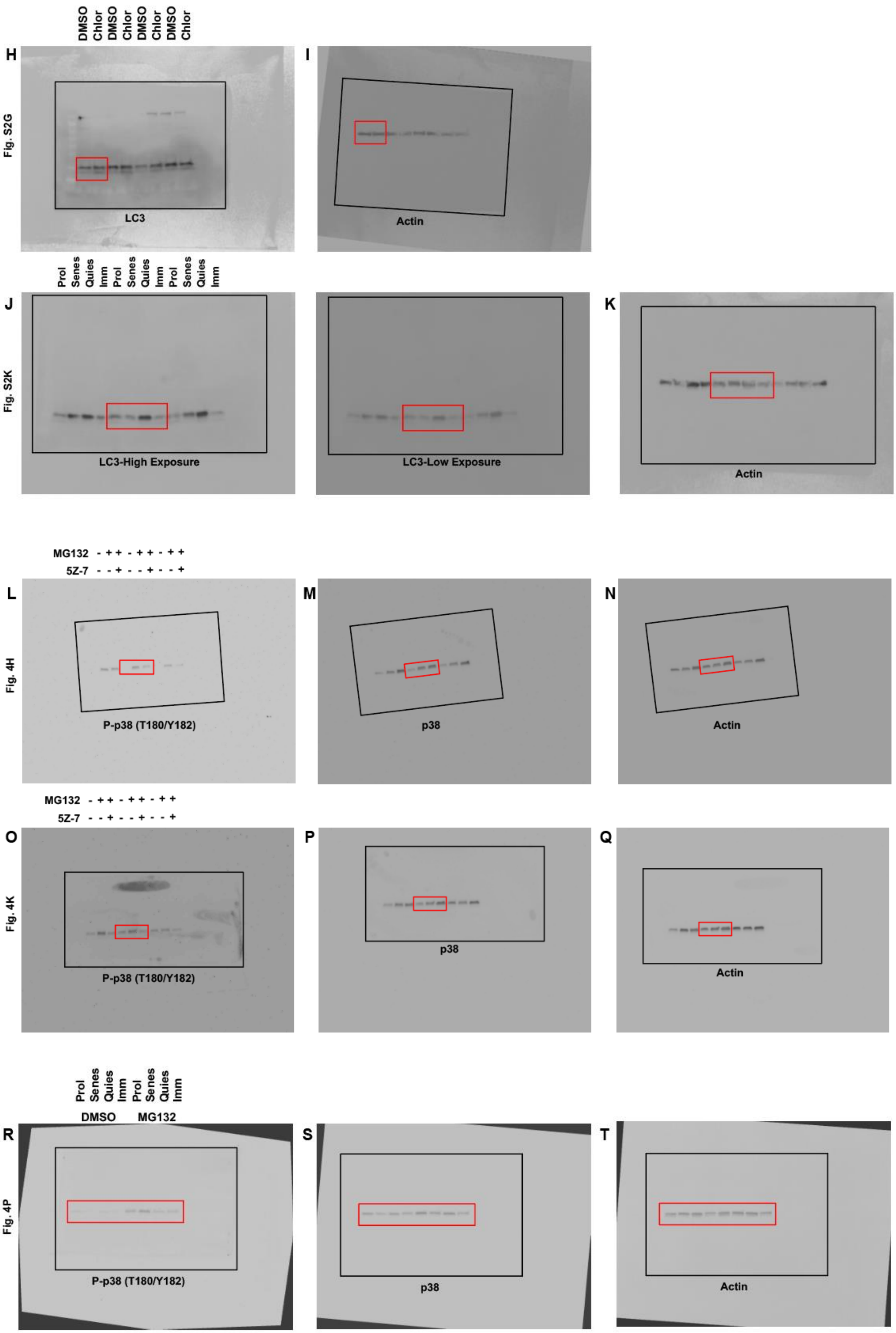

H
Fig. S2G
DMSO Chlor DMSO Chlor DMSO Chlor DMSO Chlor
LC3
I
Actin
J
Fig. S2K
Prol Senes Quies Imm Prol Senes Quies Imm Prol Senes Quies Imm
LC3-High Exposure
LC3-Low Exposure
K
Actin
L
Fig. 4H
MG132 - + + - + + - + +
5Z-7 - - + - - + - - +
P-p38 (T180/Y182)
M
p38
N
Actin
O
Fig. 4K
MG132 - + + - + + - + +
5Z-7 - - + - - + - - +
P-p38 (T180/Y182)
P
p38
Q
Actin
R
Fig. 4P
Prol Senes Quies Imm Prol Senes Quies Imm
DMSO MG132
P-p38 (T180/Y182)
S
p38
T
Actin

Figure S5 – Antibody validation and uncropped western blots. A) Fibroblasts were probed for actin, HSP90β, p38 MAPK, p-p38 MAPK (T180/Y182) and LC3 by western blot. B-C) Full-sized blots relating to Figure 2A-B. D-E) Full-sized blots relating to Figure S2A-B. F-G) Full-sized blots relating to Figure 2C-D. H-I) Full-sized blots relating to Figure S2G-J. J-K) Full-sized blots relating to Figure 2K-N. L-N) Full-sized blots relating to Figure 4H-I. O-Q) Full-sized blots relating to Figure 4J-K. R-T) Full-sized blots relating to Figure 4P-Q. All blots are shown from left to right in the order in which they were probed (left image indicates the first probe used on each blot respectively). Black box denotes edge of membrane. Red box denotes cropped region used in main figure.
